# Editorial for Special Issue “Functional Genomics and Comparative Genomics Analysis in Plants, 3rd Edition”

**DOI:** 10.3390/cimb48040394

**Published:** 2026-04-13

**Authors:** Zhen Li, Ran Su, Qiangguo Jin, Quan Zou

**Affiliations:** 1 School of Artificial Intelligence, Shenzhen University of Information Technology, Shenzhen 518172, China; lizhen5000@suit-sz.edu.cn; 2 School of Computer Software, Tianjin University, Tianjin 300350, China; ran.su@tju.edu.cn; 3 School of Software, Northwestern Polytechnical University, Xi’an 710129, China; qgking@nwpu.edu.cn; 4 Institute of Fundamental and Frontier Sciences, University of Electronic Science and Technology of China, Chengdu 611730, China

## 1. Introduction

Plant functional genomics and comparative genomics have emerged as transformative disciplines in modern plant biology, providing unprecedented insights into gene function, regulatory networks, and evolutionary trajectories across diverse species. Over the past two decades, the development of high-throughput sequencing technologies, including short-read and long-read platforms, has dramatically expanded the availability of plant genome [1] and transcriptome datasets [2], encompassing major crops, medicinal species, and wild relatives. As of 2023, more than 3500 plant genomes representing over 1500 species have been assembled and made publicly available, including hundreds produced through pan-genome projects, greatly enriching the genomic landscape for comparative and functional studies [1]. These advances have provided the foundation for systematic gene family characterization, organellar genome comparison, and stress-responsive transcriptomic profiling across a broad spectrum of plant taxa.

Despite this rapid accumulation of genomic resources, numerous challenges remain. Many ecologically, agriculturally, or medicinally important plants still lack complete and well-annotated reference genomes, and multigene families often exhibit complex duplication and divergence patterns that complicate functional inference. Comparative analyses have revealed lineage-specific variation in organelle genomes and gene content, which can influence photosynthetic efficiency, metabolic specialization, and stress adaptation [3]. Furthermore, environmental stresses, such as salinity, drought, low temperature, and heavy metals, induce extensive transcriptional reprogramming, the molecular basis of which remains poorly understood in most species [4]. Addressing these knowledge gaps is essential not only for advancing fundamental plant biology, but also for supporting crop improvement, molecular breeding, and the sustainable utilization of plant genetic resources.

Functional genomics approaches, such as genome-wide identification, transcriptome profiling, and regulatory network analysis, provide powerful tools for linking sequence information to biological function. These strategies enable systematic investigation of the molecular mechanisms underlying key processes such as nutrient acquisition, redox homeostasis, signal transduction, specialized metabolism, and reproductive development. Comparative organellar genomics further complements these efforts by uncovering structural variation, hypervariable regions, and phylogenetic patterns across species, thereby offering additional perspectives on evolutionary divergence and adaptation. At the same time, transcriptomic analyses under diverse abiotic stresses connect genomic variation with dynamic functional responses, helping to clarify the regulatory basis of plant resilience. Taken together, the integration of functional genomics, comparative genomics, and transcriptomics establishes a multidimensional framework for dissecting plant adaptation, metabolism, and development.

This Special Issue, Functional Genomics and Comparative Genomics Analysis in Plants, 3rd Edition, was curated to highlight recent advances in these fields, presenting studies that combine genome-wide identification, evolutionary analysis, and stress-responsive transcriptomics to uncover the molecular bases of adaptation, metabolic regulation, and reproductive development in diverse plant species. By leveraging these integrative approaches, the collected studies demonstrate how genomic resources can be systematically harnessed to investigate both fundamental biology and applied breeding objectives.

## 2. Review of Articles

The ten contributions in this Special Issue collectively provide a comprehensive picture of functional and comparative genomics in plants, integrating genome-wide identification, evolutionary analysis, organellar genomics, and stress-responsive transcriptomics. A unifying theme across these studies is the elucidation of gene family evolution, functional diversification, and stress adaptation in both model and non-model species.

A major focus is gene family characterization and evolutionary dynamics. Omari Alzahrani [5] systematically analyzed the AMT1 gene family in pomegranate (*Punica granatum*), identifying five members with distinct tissue-specific expression patterns and differential responses to salt stress. Phylogenetic and synteny analyses revealed conserved relationships with Arabidopsis and tomato, providing insight into the evolutionary conservation of nitrogen transporters and their potential functional roles in maintaining ammonium homeostasis under abiotic stress. Complementing this, Wang et al. [6] performed a pan-genomic analysis of mitochondrial calcium uniporter (MCU) genes across 12 tomato species, identifying 66 genes grouped into two evolutionary subfamilies. Their expression profiling under salt stress revealed distinct temporal regulation patterns, highlighting functional diversification in calcium signaling essential for stress adaptation and energy metabolism. Consistent with this broader trend, recent integrative analyses in halophytic systems have shown that comparative transcriptomic and proteomic approaches can identify lineage-specific candidate genes underlying extreme salinity tolerance, further extending functional inference beyond traditional model species [7]. Collectively, these studies underscore a pivotal shift in the field: comparative genomics is no longer limited to model organisms but is now effectively illuminating both deeply conserved mechanisms and novel, lineage-specific innovations in nutrient uptake and signal transduction.

Several studies extend this framework to stress-responsive and metabolism-related gene families. Hyun [8] examined the superoxide dismutase (SOD) family in *Platycodon grandiflorus*, demonstrating that phylogenetic relationships correlate with differential expression under oxidative stress, suggesting coordinated regulatory mechanisms for ROS detoxification. Ma et al. [9] investigated GH19 chitinase genes in Sea Island cotton, integrating expression patterns across tissues, hormone treatments, and pathogen challenge to reveal candidate genes for disease resistance. Guo et al. [10] characterized oxidosqualene cyclase (OSC) genes in *Artemisia annua*, analyzing their evolution, gene structure, cis-elements, and UV-B-responsive expression to link gene family diversification to triterpenoid biosynthesis and stress adaptation. Cui et al. [11] characterized the TaCRY gene family in wheat during seed aging, connecting gene composition and expression with physiological outcomes and developmental regulation. More broadly, recent studies have shown that calcium-mediated signaling plays an essential role in reproductive development by regulating pollen germination, maintaining pollen tube integrity, and coordinating tip-growth dynamics [12,13]. Together, these works validate that robust functional predictions can be derived from comparative genomics even in species lacking extensive genetic toolkits.

Organelle genome and comparative transcriptomic analyses provide additional layers of functional and evolutionary insight. Du et al. [14] compared chloroplast genomes across eight East Asian *Salvia* species, identifying hypervariable regions and resolving deep phylogenetic relationships, offering markers for taxonomic and evolutionary studies. Recent studies further show that transcriptome-scale regulatory analyses can uncover dynamic molecular reprogramming associated with cold adaptation and abiotic stress recovery, thereby linking genome-wide variation to physiological resilience and environmental acclimation [15,16]. These studies underscore the value of integrating genome-wide, organelle, and transcriptomic analyses to understand how molecular variation translates into functional adaptation.

Taken together, the ten articles reveal a coherent picture of integrated plant genomics research. Gene family analyses (AMT1, MCU, GH19, SOD, OSC, CaM, TaCRY) uncover both conserved and lineage-specific functional modules, highlighting the interplay between structural evolution and regulatory specialization. Organelle genome comparisons contextualize these findings in a phylogenetic framework, while transcriptomic studies link gene family dynamics to environmental and developmental responses. Collectively, the research illustrates a multidimensional approach in which evolutionary history, structural features, expression patterns, and functional relevance converge to provide a deeper understanding of plant adaptation, metabolism, and reproductive biology.

## 3. Conclusions

This Special Issue underscores the critical role of integrative functional and comparative genomics in understanding plant adaptation, metabolism, and reproduction. Studies across a wide range of species, from crops to medicinal plants, have identified key candidate genes, shed light on regulatory networks, and highlighted evolutionary patterns that contribute to stress tolerance, metabolic diversity, and reproductive processes. Importantly, insights from non-model species complement crop research, enriching our understanding of plant biology and providing actionable targets for breeding and biotechnology.

Looking forward, the path lies in deepening the integration of multi-omics datasets, such as spanning genomics, transcriptomics, epigenomics, and metabolomics, with robust functional validation. As evidenced by the studies in this issue, a particular challenge and opportunity lies in adapting these integrative frameworks for non-model species, where genetic tools are often scarce. Future efforts must prioritize bridging the gap between computational predictions derived from pan-genomes and phenotypic outcomes via advanced gene editing or transient expression systems. Pan-genome and cross-species comparisons will remain essential for understanding gene family expansion, organelle genome evolution, and stress response mechanisms. This Special Issue provides both a foundation and a roadmap for leveraging functional and comparative genomics to address current and future challenges in plant science, ultimately contributing to food security and the sustainable use of plant biodiversity.
